# Supplementary material for: Association between oral microbiome and five types of respiratory infections: a two-sample Mendelian randomization study in east Asian population
Source: Front Microbiol. 2024 Apr 10;15:1392473. doi: 10.3389/fmicb.2024.1392473 (PMC11039966; doi:10.3389/fmicb.2024.1392473)
Supplement: Supplementary file 1 [file Data_Sheet_1.zip › Supplementary Materials/MR plots of saliva/Tonsillitis/s__Streptococcus_pseudopneumoniae_O_mgs_2474/forest.pdf]

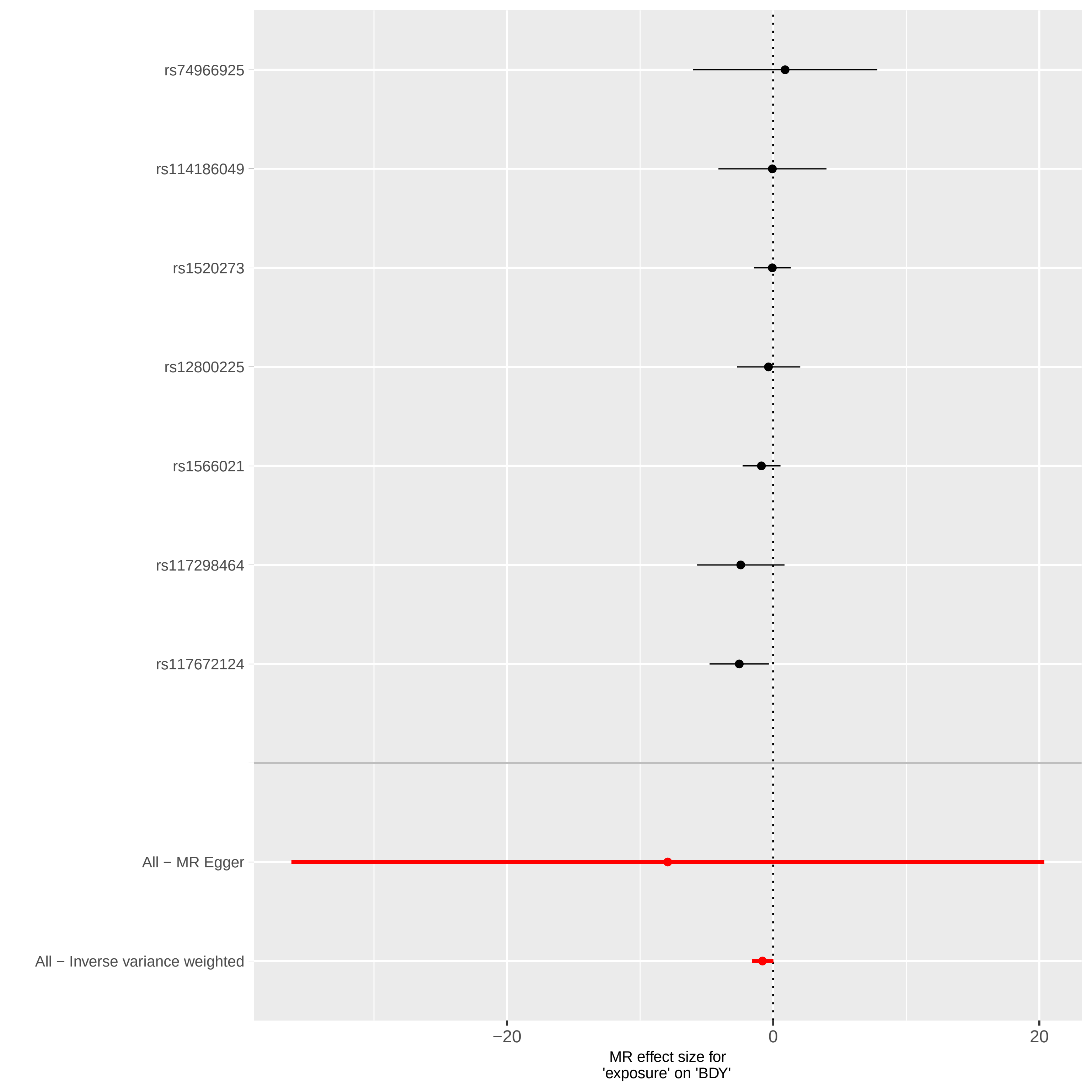
rs74966925
rs114186049
rs1520273
rs12800225
rs1566021
rs117298464
rs117672124
All − MR Egger
All − Inverse variance weighted
−20
0
20
MR effect size for
'exposure' on 'BDY'
